# Prediction of cytochrome P450-mediated drug clearance in humans based on the measured activities of selected CYPs

**DOI:** 10.1042/BSR20171161

**Published:** 2017-11-21

**Authors:** Jie Gao, Jie Wang, Na Gao, Xin Tian, Jun Zhou, Yan Fang, Hai-Feng Zhang, Qiang Wen, Lin-Jing Jia, Dan Zou, Hai-Ling Qiao

**Affiliations:** 1 Institute of Clinical Pharmacology, Zhengzhou University, Zhengzhou, China; 2 Department of Histology and Embryology, Henan Medical College, Zhengzhou, China

**Keywords:** cytochrome P450 enzymes, drug clearance, in vitro, in vivo

## Abstract

Determining drug-metabolizing enzyme activities on an individual basis is an important component of personalized medicine, and cytochrome P450 enzymes (CYPs) play a principal role in hepatic drug metabolism. Herein, a simple method for predicting the major CYP-mediated drug clearance *in vitro* and *in vivo* is presented. Ten CYP-mediated drug metabolic activities in human liver microsomes (HLMs) from 105 normal liver samples were determined. The descriptive models for predicting the activities of these CYPs in HLMs were developed solely on the basis of the measured activities of a smaller number of more readily assayed CYPs. The descriptive models then were combined with the Conventional Bias Corrected *in vitro*–*in vivo* extrapolation method to extrapolate drug clearance *in vivo.* The V_max_, K_m_, and CL_int_ of six CYPs (CYP2A6, 2C8, 2D6, 2E1, and 3A4/5) could be predicted by measuring the activities of four CYPs (CYP1A2, 2B6, 2C9, and 2C19) in HLMs. Based on the predicted CL_int_, the values of CYP2A6-, 2C8-, 2D6-, 2E1-, and 3A4/5-mediated drug clearance *in vivo* were extrapolated and found that the values for all five drugs were close to the observed clearance *in vivo*. The percentage of extrapolated values of clearance *in vivo* which fell within 2-fold of the observed clearance ranged from 75.2% to 98.1%. These findings suggest that measuring the activity of CYP1A2, 2B6, 2C9, and 2C19 allowed us to accurately predict CYP2A6-, 2C8-, 2D6-, 2E1-, and 3A4/5-mediated activities *in vitro* and *in vivo* and may possibly be helpful for the assessment of an individual’s drug metabolic profile.

## Introduction

As the principal class of hepatic drug metabolizing enzymes, CYPs play a critically important role in the biosynthesis and degradation of endogenous compounds and the metabolism of drugs and environmental procarcinogens [1]. Interindividual variation in drug metabolism, which encompasses genetic polymorphisms of CYPs [2,3], smoking [2,3], drinking [2,3], age [4], and gender [5,6], has a substantial impact on individual drug safety and efficacy, raising a challenge to guide individualized medicine.

It is usually agreed that patient differences in pharmacokinetics largely result from differences in the activities of an individual’s drug metabolizing enzymes, and is the chief reason for different responses to drugs [7–9]. Therefore, determining the drug metabolizing enzyme activities of an individual is an important prerequisite for personalized medicine. In addition, the area under the blood concentration–time curve and the steady-state blood concentration depend on drug clearance *in vivo* (CL_H_) considered to be directly related to the pharmacological effects or adverse effects of a drug. Therefore, having information on the CL_H_ is a necessary condition for individual dosage regimens.

A multitude of different CYPs share similar physical and molecular characteristics, are colocalized on the cytoplasmic side of the endoplasmic reticulum [10,11], and coordinately carry out the biosynthesis and degradation of endogenous steroids, lipids, and vitamins as well as many exogenous substances [12–15]. In addition, a substantial degree of correlation among microsomal CYP activities was reported in two previous studies [4,16]. Another study found that the expression levels of almost all xenobiotic-metabolizing genes were strongly correlated with each other at the mRNA level [17]. Our previous studies also have shown that a high degree of correlation existed at the mRNA and protein expression levels of CYPs [18]. These correlations among CYPs at the protein, mRNA, and activity levels suggest that descriptive models based on multiple linear regression might be developed to predict the activities of some CYPs solely on the basis of measured activities of a smaller number of more readily assayed CYP enzymes.

*In vitro*–*in vivo* extrapolation (IVIVE) is an important method for estimating the *in vivo* clearance of drugs based on the *in vitro* intrinsic clearance data determined in human liver microsomes [19]. The IVIVE method is useful in providing insight into the rate of elimination of drugs from the body and helping physicians make dosage adjustments. Recently, to predict the *in vivo* clearance for CYPs more accurately, we introduced correction coefficients into the IVIVE method based on the study of Halifax and Houston, who developed the conventional bias corrected *in vitro*–*in vivo* extrapolation (CBC-IVIVE) method [2,20]. Combining descriptive models and CBC-IVIVE might allow us to accurately predict total CYP-mediated drug clearance *in vivo* based on the measured activities of a few CYPs using a small quantity of liver tissue.

Herein, we obtained 105 liver tissue samples derived from 123 liver samples [21] taken from normal tissue adjacent to surgical biopsies, which allowed us to measure the activities of ten CYPs in each sample and provided the foundation for the development of descriptive models that could be used to estimate the activities of six CYPs by actually measuring four CYP activities. To strengthen the clinical values, the CL_H_ of probe drugs for six CYPs were extrapolated using CBC-IVIVE method, and the extrapolation accuracy was evaluated.

## Materials and methods

### Chemicals and reagents

All probe drugs (phenacetin, coumarin, bupropion, paclitaxel, tolbutamide, omeprazole, dextromethorphan, chlorzoxazone, and midazolam) and one metabolite (acetaminophen) were purchased from the National Institute for the Food and Drug Control (China). Other metabolites (7-OH-coumarin, 4-OH-bupropion, 6-OH-paclitaxel, 4-OH-tolbutamide, 4-OH-omeprazole, 3-methoxymorphinan, 6-OH-chlorzoxazone, and 1-OH-midazolam) were obtained from Toronto Research Chemicals, Inc. (Canada). Reduced nicotinamide adenine dinucleotide phosphate and horse cytochrome *C* were obtained from Solarbio Science and Technology co. (China). Methanol and acetonitrile were HPLC grade and were purchased from Siyou Chemical Reagent Co. (China).

### Human liver microsomes (HLMs)

As reported recently [21], 105 liver samples were selected from 123 liver samples obtained from patients undergoing liver surgery during 2012 and 2014 at the First Affiliated Hospital of Zhengzhou University, the People’s Hospital of Henan Province, and the Tumors’ Hospital of Henan Province. The present study was conducted according to the World Medical Association Declaration of Helsinki, authorized by the ethics committees of Zhengzhou University (Zhengzhou, China), and written informed consent was obtained from each volunteer. All experiments were performed in accordance with the approved guidelines of the ethics committees of Zhengzhou University.

Detailed information [gender, age, smoking, drinking, body weight (BW), and medical diagnosis] for each patient was well documented. In accord with previous research [22], the smokers were defined as those who smoke 11 or more cigarettes per day and non-smokers were defined as those who smoke less than 11 cigarettes per day or never smoked; drinkers were defined as those who have consumed alcohol 2–3 times or more per week, and non-drinkers were defined as those who have consumed alcohol less than two times per week or never drunk. All patients were subjected to routine anesthetic use for the procedure and had no history of exposure to known CYP-inducing or inhibiting agents. Samples from normal livers were collected, with liver health confirmed by liver function tests, histopathological analysis, and imaging examination by ultrasonography or CT. Following extraction, liver samples were immediately frozen and stored in liquid nitrogen until use. As described recently [23], HLMs were prepared by differential centrifugation and stored at −80°C until analysis. Microsomal protein content was determined by the Bradford method [24].

### Measurement of ten CYP-mediated metabolic activities *in vitro*

According to the recent methods [25], ten CYP-mediated metabolic activities were measured in individual assays by incubating HLMs (0.3 mg protein/ml for CYP1A2, 2A6, and 2E1; 0.2 mg protein/ml for CYP2D6 and 3A4/5; 0.5 mg protein/ml for CYP2B6, 2C8, 2C9, and 2C19), 100 mM phosphate buffer (pH 7.4), and 1 mM reduced nicotinamide adenine dinucleotide phosphate with seven or eight concentrations of substrate (6.25–800 μM for phenacetin, 0.156–20 μM for coumarin, 7.8–500 μM for bupropion, 2.5–80 μM for paclitaxel, 31.25–2000 μM for tolbutamide, 3.9–500 μM for omeprazole, 0.625–960 μM for dextromethorphan, 7.8–1000 μM for chlorzoxazone, and 0.39–50 μM for midazolam). The mixtures were preincubated for 5 min at 37°C. Optimal incubation times were as follows: 30 min for phenacetin *O*-deethylation, coumarin 7-hydroxylation, and chlorzoxazone 6-hydroxylation; 60 min for bupropion 4-hydroxylation and tolbutamide 4-hydroxylation; 90 min for omeprazole 5-hydroxylation; 120 min for paclitaxel 6-hydroxylation; 20 min for dextromethorphan *O*-demethylation; and 5 min for midazolam 1′-hydroxylation. Incubation conditions ensured linear metabolite formation with respect to reaction time and protein content.

Reactions were terminated by adding 20 μl of ice-cold acetonitrile (phenacetin, omeprazole, and midazolam), 1 ml of ethylacetate (paclitaxel and chlorzoxazone), or 10 μl of perchloric acid (coumarin, bupropion, tolbutamide, and dextromethorphan). Substrate metabolites were identified by HPLC-UV (acetaminophen, 4-OH-bupropion, 6-OH-paclitaxel, 4-OH-tolbutamide, 4-OH-omeprazole, 6-OH-chlorzoxazone, and 1-OH-midazolam) or HPLC-FLD (7-OH-coumarin and 3-methoxymorphinan). The detailed description of analytical methods for the substrate metabolites is provided in Supplementary Table S1. The Michaelis–Menten constant (K_m_) and maximum reaction rate (V_max_) of each CYP were determined by nonlinear regression analysis using GraphPad Prism 5.04 (GraphPad Inc., La Jolla, CA, U.S.A.). Intrinsic clearance (CL_int_) was calculated based on the ratio of V_max_-to-K_m_.

### Prediction of six CYP-mediated metabolic activities *in vitro*

#### Development of descriptive models

Predictive descriptive models for each kinetic parameter (V_max_, K_m_, and CL_int_) for each CYP can be developed using SPSS 17.0 (SPSS Inc., Chicago, IL, U.S.A.), as follows: First, each kinetic parameter (V_max_, K_m_, and CL_int_) of the ten CYPs was treated as a dataset, which then generated three datasets. For each training set, the data of one CYP were set as the dependent variable and the datasets of the other CYPs were set as independent variables, from which a multiple linear regression model was developed by a stepwise method (criteria: probability of *F* to enter was ≤0.05, probability of *F* to remove was ≥0.10). For every model, the coefficient of determination (*R*^2^) and adjusted coefficient of determination (*R*^2^_ad_) were calculated.

#### Prediction of activities for six CYPs

A full-scale analysis of all multiple linear regression equations was determined based on the *ab initio* assumption that CYP activity was independent of other enzyme activities. Using the equations generated and refined above, it was found that the activities of six CYPs (CYP2A6, 2C8, 2D6, 2E, and 3A4/5) could be predicted based on the measured activities of four CYPs (CYP1A2, 2B6, 2C9, and 2C19).

#### Accuracy of predicted *CL_int_*

As only the CL_int_ was used to extrapolate the CL_H_, the accuracy of the predicted CL_int_ was evaluated. The normality of the data distribution was first assessed using the method of Kolmogorov–Smirnov and Shapiro–Wilk. Because most datasets were not normally distributed, the overall accuracy of prediction was explored using Mann–Whitney *U* test to compare the different distribution between the measured and predicted CL_int_. In order to estimate the accuracy of prediction for each case, the ratio of predicted CL_int_-to-measured CL_int_ for every individual was calculated and a 2-fold precision limit was set.

### Extrapolation of six CYP-mediated drug clearance values *in vivo*

#### CBC-IVIVE method

According to previous reports [2,20], the equation of the CBC-IVIVE is

$${\mathrm{CL}}_{H}\;=\;\mathrm{CC}\;\times \;\frac{Q_{H}\;\times \;{\mathrm{CL}}_{\mathrm{int}}\;\times \;\mathrm{MPPGL}\;\times \;(\mathrm{LW}/\mathrm{BW}\;\times \;f_{u,p}/R_{B})}{Q_{H}\;+\;{\mathrm{CL}}_{\mathrm{int}}\;\times \;\mathrm{MPPGL}\;\times \;(\mathrm{LW}/\mathrm{BW}\;\times \;f_{u,p}/R_{B})}$$

Where Q_H_ (ml/min) was determined as 24.5% [26] of the cardiac output. Cardiac output originated from data for normal Han Chinese females (n=805) and males (n=783) [27]. Microsomal protein per gram of liver (MPPGL) contents were determined using cytochrome P450 oxidoreductase activity as measured in homogenates and microsomes obtained from the same liver tissue sample [28,29]; The liver weight (LW) was calculated by multiplying the liver volume by the liver density, where liver volume (ml) = 12.5 × BW + 536.4 [30] and the liver density was 1.001 g/ml [31]. The correction coefficient (CC), the plasma unbound fraction (f_u,p_), and blood-to-plasma concentration ratio (R_B_) of each probe drug for six CYPs were obtained from literature [2,32–36].

#### Extrapolation-based measured or predicted CL_int_

According to the CL_int_ (measured or predicted above) and other parameters, CYP2A6, 2C8, 2D6, 2E, and 3A4/5-mediated CL_H_ values were extrapolated (referred to as predicted CL_H_ and CL’_H_) using the CBC-IVIVE strategy.

#### Accuracy of predicted CL_H_ and CL’_H_

The overall accuracy of the predictions was assessed from the average fold-error (AFE) and the different distribution between CL_H_ and CL’_H_, while the individual accuracy was assessed based on the individual fold-error (IFE). Because most datasets were not normally distributed, the different distribution between CL_H_ and CL’_H_ was explored using Mann–Whitney *U* test. A 2-fold precision limit corresponds to 0.5–2 of AFE or IFE values, where, $\text{AFE}=10^{1/N[\Sigma \log(predicted\;mean/observed\;overall\;mean)]}$, $\text{IFE}=10^{1/N[\Sigma \log(predicted\;individual\;value/observed\;overall\;mean)]}$ [2]. N refers to the number of separate reports in the literature concerning intravenous drug clearance, except for chlorzoxazone.

### Statistical analyses

Statistical analysis was performed using SPSS 17.0 software (SPSS Inc., Chicago, IL, U.S.A.), and a *P*-value < 0.05 was considered to be statistically significant (two-tailed). All graphs were generated using the Adobe Photoshop CC 2014 and GraphPad Prism 5.04 software package (GraphPad Inc., La Jolla, CA, U.S.A.).

## Results

### Measurement of ten CYP-mediated metabolic activities *in vitro*

As shown in Table 1, the basic clinical characteristics of human liver samples were collected from 105 subjects. Among all subjects, women in a majority of cases, over half the subjects were between 45 and 59 years old. Most subjects had no smoking or drinking history. Most of the subjects experienced liver hemangioma. All subjects received only regular anesthetics and had no history of exposure to known CYP-inducing or -inhibiting agents.

**Table 1:** The basic clinical characteristics of human liver samples (n=105)

Variables	Group	Number (percent)
Gender	Male	37 (35.2%)
	Female	68 (64.8%)
Age (years)	<44	35 (33.3%)
	45–59	56 (53.3%)
	60–74	13 (12.4%)
	>75	1 (1.0%)
Smoking	Yes	12 (11.9%)
	No	89 (88.1%)
Drinking	Yes	12 (11.9%)
	No	89 (88.1%)
Medical diagnosis	Liver hemangioma	84 (80.0%)
	Cholelithiasis	9 (8.6%)
	Metastatic carcinoma	8 (7.6%)
	Gallbladder cancer	4 (3.8%)

The above normal Chinese liver samples were used to measure the ten CYPs (CYP1A2, 2A6, 2B6, 2C8, 2C9, 2C19, 2D6, 2E1, and 3A4/5)-mediated metabolic activities *in vitro* using probe substrate metabolism assays. The activities were described as kinetic parameters (V_max_, K_m_, and CL_int_), and the results are presented in Table 2.

**Table 2:** The V_max_, K_m_, and CL_int_ of ten CYPs in human liver microsomes (n=105)

CYPs	V_max_ (pmol/min/mg protein)	K_m_ (μM)	CL_int_ (μl/min/mg protein)
1A2	754.9(94.9–3154.0)	54.7(4.7–181.6)	14.5(2.8–67.2)
2A6	354.4(3.7–3295.0)	2.3(0.8–10.0)	145.0(1.2–544.7)
2B6	53.3(12.8–333.5)	73.4(17.1–393.3)	0.77(0.13–5.22)
2C8	37.5(2.8–174.6)	14.3(7.0–38.9)	2.70(0.09–6.19)
2C9	256.2(83.8–454.8)	219.2(101.2–555.3)	1.17(0.17–4.18)
2C19	103.9(2.3–381.4)	59.7(20.6–198.3)	1.91(0.01–7.46)
2D6	113.3(23.5–1041.0)	28.9(6.5–260.6)	3.5(0.2–39.5)
2E1	532.1(163.1–1982.0)	52.5(27.1–177.2)	10.5(1.9–39.0)
3A4/5	788.0(69.4–5035.0)	1.9(0.4–10.2)	464.6(8.3–1673.5)

The *K*_m_ and *V*_max_ of each CYP were determined by nonlinear regression analysis using GraphPad Prism 5.04. The CL_int_ was calculated based on the ratio of *V*_max_-to-*K*_m_. Data are shown as median and range.

### Prediction of six CYP-mediated metabolic activities *in vitro*

#### Development of the descriptive models

The descriptive models were developed using a multiple linear regression method, based on measured values. The results show that the descriptive models of V_max_ and CL_int_ of all ten CYPs and K_m_ of six CYPs (CYP1A2, 2B6, 2C9, 2C19, and 3A4/5) could be developed, and the essential structures of these models consisted of the measured V_max_, CL_int_, and K_m_ of CYPs (data not shown). In order to predict activities of several CYPs based on known CYP activities, the principle that the numbers of CYPs were known as little as possible was upheld to analyze all multiple linear regression equations carefully. The results indicate that the six CYPs (CYP2A6, 2C8, 2D6, 2E1, and 3A4/5)-mediated metabolic activities *in vitro* could be predicted if the activities of four CYPs (CYP1A2, 2B6, 2C9, and 2C19) measured *in vitro* were known. Table 3 summarizes the regression equations and statistical parameters of these models.

**Table 3:** The descriptive models for six CYPs in human liver microsomes

Parameters	Regression equation	Known	*F*	*P*	*R*^2^	*R*^2^_ad_
V_max_ (pmol/min/mg protein)	2A6 = 104.899 + 2.292 × 2C19	2C19	21.559	1.015E−05	0.173	0.165
	2D6 = 10.698 + 0.481 × 2C9	2C9	12.655	5.677E−04	0.109	0.101
	2E1 = 362.868 + 0.290 × 1A2	1A2	25.152	2.217E−06	0.196	0.188
	2C8 = -6.784 + 0.158 × 2C9 + 0.143 × 2B6	2C9, 2B6	24.813	1.656E−09	0.327	0.314
	3A4/5 = 106.151 + 9.416 × 2B6 + 2.504 × 2C19	2B6, 2C19	21.209	1.985E−08	0.294	0.280
K_m_ (μM)	3A4/5 = 2.941 - 0.013 × 1A2	1A2	7.641	6.762E−03	0.069	0.060
CL_int_ (μl/min/mg protein)	2A6 = 120.384 + 9.662 × 2C19	2C19	4.381	3.881E−02	0.041	0.031
	2C8 = 1.693 + 0.862 × 2C9	2C9	31.661	1.590E−07	0.235	0.228
	2D6 = 3.325 + 1.438 × 2B6	2B6	5.250	2.399E−02	0.048	0.039
	2E1 = 6.414+1.218×2C19+0.143×1A2	2C19, 1A2	11.108	4.319E−05	0.179	0.163
	3A4/5 = 407.070 + 77.635 × 2C19 - 8.003 × 1A2 + 87.331 × 2B6	2C19, 1A2, 2B6	7.257	1.858E−04	0.177	0.153

*R*^2^, coefficient of determination; *R*^2^_ad_, adjusted coefficient of determination.

#### Prediction of activities for six CYPs

Of note, prediction of some CYP-mediated metabolic activities in vitro did not require knowledge of all four CYPs activities; some activities could be predicted on just one or two known CYP activities. More specifically, the V_max_ of CYP2A6 could be predicted based on the V_max_ of CYP2C19, the V_max_ of CYP2D6 could be predicted by the V_max_ of CYP2C9, the V_max_ of CYP2E1 could be predicted by the V_max_ of CYP1A2, the V_max_ of CYP2C8 could be predicted based on the V_max_ values of CYP2C9 and 2B6, and the V_max_ values of CYP3A4/5 could be predicted based on the V_max_ values of CYP2B6 and 2C19. For CL_int_, CYP2A6 could be predicted based on CYP2C19, CYP2C8 could be predicted based on CYP2C9, CYP2D6 could be predicted based on CYP2B6, CYP2E1 could be predicted based on CYP1A2 and 2C19, CYP3A4/5 could be predicted based on CYP1A2, 2B6, and 2C19. For K_m_, although some descriptive models of CYPs could not be developed, the K_m_ values of most CYPs could be calculated as their respective V_max_ divided by corresponding CL_int_.

Taken together, the activity of CYP2A6 could be predicted based on the activity of CYP2C19, CYP2C8 and 2D6 could be predicted based on CYP2B6 and 2C9, CYP2E1 could be predicted based on CYP2C19 and 1A2, CYP3A4/5 could be predicted based on CYP1A2, 2B6, and 2C19. In short, the prediction for six CYPs needed 1–3 measured CYP values. The median values, ranges, and 95% prediction intervals of predicted V_max_, K_m_, and CL_int_ for CYP2A6, 2C8, 2D6, 2E1, and 3A4/5 are summarized in Table 4. The biggest individual variations in predicted CL_int_ took place in the CYP3A4/5, reaching to 4.1-fold, followed by that of CYP2D6, CYP2C8, CYP2E1, and CYP2A6, demonstrating the fold-change of 3.1, 2.9, 2.6, and 1.6 respectively.

**Table 4:** The predicted V_max_ (pmol/min/mg protein), K_m_ (μM), and CL_int_ (μl/min/mg protein) for six CYPs in human liver microsomes (n=105)

CYPs	Parameter	Median	Range	95%PI
2A6	V_max_	344.4	110.3–979.1	154.5–857.4
	K_m_	2.5	0.9–5.4	1.3–5.1
	CL_int_	138.9	120.5–192.5	122.9–176.7
2C8	V_max_	42.3	8.4–84.0	12.7–76.9
	K_m_	15.0	4.5–30.3	6.8–23.9
	CL_int_	2.7	1.8–5.3	1.9–4.4
2D6	V_max_	133.9	51.0–229.5	52.7–216.5
	K_m_	29.6	10.0–49.0	11.3–46.4
	CL_int_	4.4	3.5–10.8	3.6–8.0
2E1	V_max_	581.8	390.3–1227.5	421.8–1115.5
	K_m_	53.9	30.8–93.2	36.4–71.9
	CL_int_	11.0	7.2–18.7	7.9–17.3
3A4/5	V_max_	897.7	302.7–3493.1	434.3–1837.2
	K_m_	1.8	0.7–4.6	1.0–4.3
	CL_int_	507.5	235.9–958.3	278.6–871.5

95%PI, 95% prediction interval. The values of V_max_ and CL_int_ for six CYPs were predicted using the descriptive models summarized in Table 1. Because descriptive models of K_m_ for most CYPs could not be developed, K_m_ values of six CYPs were calculated as their respective V_max_ divided by corresponding CL_int_.

#### Accuracy of predicted CL_int_

**Figure 1:**
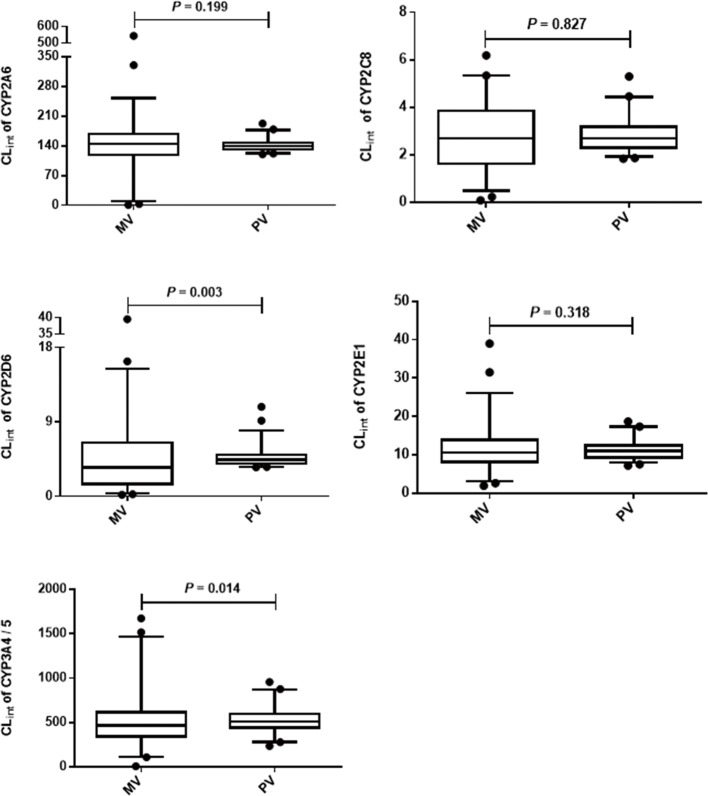
The overall accuracy of predicted CL_int_ of CYP2A6, 2C8, 2D6, 2E1, and 3A4/5 The data are presented as the 2.5–97.5 percentile; Abbreviations: MV, measured value; PV, predicted value determined by the descriptive model. Mann–Whitney *U* was used to evaluate the difference between predicted and measured values

Because only the CL_int_ was used to extrapolate the clearance *in vivo*, the accuracy of predicted CL_int_ was estimated. For the overall accuracy of prediction, there were no apparent statistical differences in the measured and predicted CL_int_ values of CYP2A6, 2C8, and 2E1 (Figure 1). Nevertheless, the predicted CL_int_ values of CYP2D6 (*P*=0.003) and 3A4/5 (*P*=0.014) were significantly higher than the measured values. The ranges of predicted CL_int_ for all CYPs were smaller than the measured data. In other words, the predicted CL_int_ values narrowed the interindividual variation of measured values, which might influence the accuracy of the predicted clearance *in vivo*.

To estimate the accuracy of predicted CL_int_ for each case, the ratio of predicted CL_int_-to-measured CL_int_ for every individual was calculated, and results were presented in Table 5. For most subjects (87.6 percent), the predicted CL_int_ for coumarin and paclitaxel was very close to the measured CL_int_. However, for nearly half the subjects (47.6 percent) the predicted CL_int_ of dextromethorphan was close to the measured CL_int_.

**Table 5:** The individual accuracy of predicted CL_int_ (the ratio of predicted CL_int_-to-measured CL_int_) of CYP2A6, 2C8, 2D6, 2E1, and 3A4/5 (*n*=105)

CYP	Probe drug	Median	Range	Within a 2-fold error (*n*, %)
2A6	Coumarin	0.97	0.26–114.7	92 (87.6%)
2C8	Paclitaxel	1.02	0.30–36.3	92 (87.6%)
2D6	Dextromethorphan	1.36	0.13–27.4	50 (47.6%)
2E1	Chlorzoxazone	1.06	0.42–4.96	90 (85.7%)
3A4/5	Midazolam	1.17	0.35–33.7	87 (82.9)

### Extrapolation of six CYP-mediated drug clearance values *in vivo*

#### CBC-IVIVE

The parameters for the equations of the CBC-IVIVE for five probe drugs are based on previous reports and listed in Table 6. Of note, the Q_H_, MPPGL, LW, and BW were 1259.3 (1205.4–1629.3) ml/min, 39.6 (9.9–127.9) mg/g, 1337.2 (912.3–1688.1) g, and 64.0 (30.0–92.0) kg respectively. The variable degrees of the other three parameters were relatively lower, with only BW variations reaching 3-fold.

**Table 6:** The parameters in the equations of the CBC-IVIVE and clearance *in vivo* (CL_H_, ml/min) of five probe drugs

CYP	Probe drug	Parameters in the Equation of the CBC-IVIVE*			Observed CL_H_	Predicted CL_H_ (n=105)	Predicted CL’_H_ (n=105)
		CC	f_u,p_	R_B_			
2A6	Coumarin	5.369 [2]	0.055 [2]	1 [2]	1602.5 ± 547.9 [37]	1602.5 ± 748.2	1692.3 ± 622.8
2C8	Paclitaxel	18.938 [2]	0.098 [32]	0.69 [33]	496.4 ± 210.5 [38–43]	422.2 ± 328.6	413.6 ± 226.8
2D6	Dextromethorphan	35.791 [2]	0.500 [34,35]	0.55 [34,35]	6471.7 ± 5596.7 [44]	6471.7 ± 5816.5	7016.1 ± 3202.7
2E1	Chlorzoxazone	4.152 [2]	0.028 [34]	0.55 [34]	131.4 ± 40.1 [45–49]	131.4 ± 97.4	130.1 ± 69.3
3A4/5	Midazolam	0.540 [2]	0.042 [34,36]	0.54 [34,36]	426.7 ± 95.4 [36,50,51]	403.8 ± 128.3	426.2 ± 95.1

* The equation of the CBC-IVIVE (conventional bias-corrected *in vitro*–in vivo extrapolation) was ${\mathrm{CL}}_{H}\;=\;\mathrm{CC}\;\times \;\frac{Q_{H}\;\times \;{\mathrm{CL}}_{\mathrm{int}\;}\times \;\mathrm{MPPGL}\;\times \;(\mathrm{LW}/\mathrm{BW}\;\times \;f_{u,p}/R_{B})}{Q_{H}\;+\;{\mathrm{CL}}_{\mathrm{int}\;}\times \;\mathrm{MPPGL}\;\times \;(\mathrm{LW}/\mathrm{BW}\;\times \;f_{u,p}/R_{B})}$. Abbreviations: BW, body weight; CC, correction coefficient; CL_int_, intrinsic clearance; LW, liver weight; MPPGL, microsomal protein per gram of liver; Q_H_, hepatic blood flow. Observed CL_H_ was the clearance *in vivo* reported in the literature. Using the CBC-IVIVE method, predicted CL_H_ was calculated based on measured CL_int_, and predicted CL’_H_ was calculated based on predicted CL_int_.

#### Extrapolation based on measured CL_int_

According to the measured CL_int_ in HLMs, the values of CL_H_ for coumarin, paclitaxel, dextromethorphan, chlorzoxazone, and midazolam which were probe substrates for CYP2A6, 2C8, 2D6, 2E1, and 3A4/5 were extrapolated using the CBC-IVIVE strategy. The predicted and observed CL_H_ for all five drugs are shown in Table 6. The mean values for the predicted and observed CL_H_ of coumarin, dextromethorphan, and chlorzoxazone were the same, but the predicted CL_H_ showed larger individual variations. The mean values for the predicted CL_H_ of paclitaxel and midazolam were relatively smaller than observed CL_H_, but the predicted values showed obvious variations. The individual variation in the values of CL_H_ for dextromethorphan was the largest, with the coefficient of variation (CV) of 89.9%, followed by that of paclitaxel, chlorzoxazone, and coumarin, demonstrating the CV of 77.8%, 74.1%, and 46.7 respectively. Compared with other drugs, CV of midazolam CL_H_ was much lower but still achieving 31.8%.

#### Extrapolation based on predicted CL_int_

According to the CL_int_ that was predicted above and other parameters, the values of CL’_H_ for coumarin, paclitaxel, dextromethorphan, chlorzoxazone, and midazolam were extrapolated using the CBC-IVIVE strategy. As shown in Table 6, considering the values of CL’_H_ for all five drugs, the mean value and SD of CL’_H_ for midazolam matched best with its observed CL_H_, while the mean value CL’_H_ for dextromethorphan matched poorly with its observed CL_H_. The individual variation in the values of CL’_H_ for paclitaxel was the largest, with the CV of 54.8%, followed by that of chlorzoxazone, dextromethorphan, coumarin, and midazolam, demonstrating the CV of 53.3%, 45.6%, 36.8%, and 22.3% respectively. Compared with the individual variations in the values of CL_H_ for all five drugs, the individual variations in the values of CL’_H_ were much smaller.

#### Accuracy of predicted CL_H_ and CL’_H_

**Figure 2:**
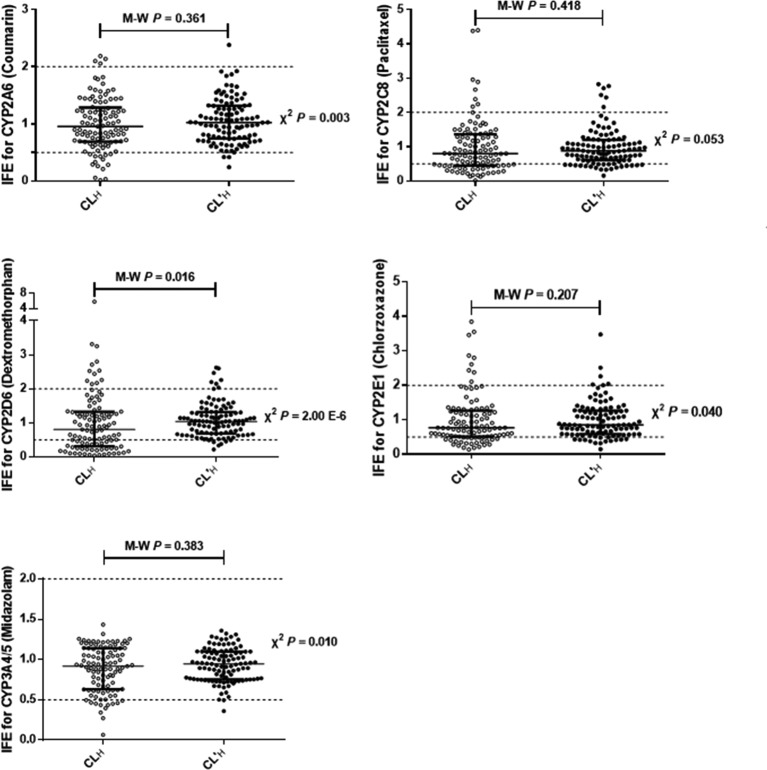
The accuracy of predicted CL_H_ or CL’_H_ for coumarin, paclitaxel, dextromethorphan, chlorzoxazone, and midazolam which are the probe substrates of CYP2A6, 2C8, 2D6, 2E1, and 3A4/5 respectively (*n*=105) IFE is the individual fold-error. CL_H_ is the *in vivo* clearance or hepatic clearance. Using the conventional bias-corrected *in vitro*–*in vivo* extrapolation method, predicted CL_H_ was calculated based on measured CL_int_, and predicted CL’_H_ was calculated based on predicted CL_int_. The black horizontal solid line represents the median value and interquartile range. $\text{IFE}=10^{1/N[\Sigma \log(predicted\;individual\;value/observed\;overall\;mean)]}$. Mann–Whitney *U* test was used to evaluate the differences between the IVE of CL_H_ and CL’_H_. Cross tabs with *χ*^2^ tests for independence analyses revealed that the difference between the number (percentage) was within 2-fold error of the observed CL_H_ in the CL_H_ and CL’_H_ groups.

To evaluate the extrapolation performance, the accuracy of the predicted CL_H_ and CL’_H_ values for drugs were compared with the observed CL_H_ (Figure 2). Whether predicted CL_H_ or CL’_H_, the average fold-error (AFE) values for five drugs were adjacent to 1, which demonstrated that the extrapolation performance was accurate. Of note, the values of the predicted CL’_H_ based on predicted CL_int_ for all five drugs were closer to the observed CL_H_ than the predicted CL_H_ based on measured CL_int_. The predicted CL_H_ value reduces the difference between individuals, resulting that a more accurate estimate of CL_H_ than measured CL_int_.

From another angle to analyze this problem, the distribution of the individual fold-error (IFE) of predicted CL_H_ and CL’_H_ for all five drugs were explored (Figure 2). The results support the distributions of IFE for coumarin, paclitaxel, chlorzoxazone, and midazolam in the CL_H_ and CL’_H_ groups and showed no significant differences, while there was enough evidence to say that the distributions of IFE for dextromethorphan in the CL_H_ and CL’_H_ groups were significantly different.

To test the accuracy of predicted CL_H_ and CL’_H_ for each individuals, the IFE was also calculated. The predicted CL’_H_ value for midazolam matched most closely with its observed CL_H_, for which 103 (98.1%) of the cases were within a 2-fold error range (Figure 2). After midazolam, the CL’_H_ value for coumarin, also matched best with its clearance *in vivo*, for which 101 (96.2%) of the cases were within a 2-fold error range. Meanwhile, the accuracy of predicted CL’_H_ of dextromethorphan, chlorzoxazone, and paclitaxel for each individuals was also quite outstanding, for which 90 (85.7%), 85 (81.0%), and 79 (75.2%) of the cases respectively, were within a 2-fold error range. While among the five drugs, the predicted CL_H_ value for midazolam matched most closely with its observed CL_H_, for which 93 (88.6%) of the cases were within a 2-fold error range. The number (percent) of predicted CL_H_ for coumarin, chlorzoxazone, paclitaxel, and dextromethorphan that fell within 2-fold of the observed CL_H_ were 87 (82.9%), 71 (67.6%), 65 (61.9%), and 58 (55.2%) respectively.

For all five drugs the percentage of predictions that fell within 2-fold of the observed CL_H_ were different, which used different sources of CL_int_ (measured or predicted). Cross tabs with χ^2^ tests for independence analyses revealed that the values of predicted CL’_H_ for coumarin, dextromethorphan, chlorzoxazone, and midazolam were more accurate than those of predicted CL_H_, while the prediction accuracy for paclitaxel was not significantly different between the predicted CL_H_ and CL’_H_ using different sources of CL_int_.

## Discussion

The purpose of the present study was to determine the drug clearance values for six CYPs *in vivo* and *in vivo* based on measured activities of four CYPs in HLMs. Descriptive models for predicting the activities of CYPs in HLMs were developed first and the activities of six CYPs (CYP2A6, 2C8, 2D6, 2E1, and 3A4/5) could be predicted by actually measuring the activities of four CYPs (CYP1A2, 2B6, 2C9, and 2C19). In addition, based on the predicted CL_int_, the clearances for CYP2A6, 2C8, 2D6, 2E1, and 3A4/5 *in vivo* were extrapolated using the CBC-IVIVE method [2,20]. We found that the extrapolation performance was accurate with the AFE values of 1.06, 0.98, 1.08, 0.99, and 0.93 while the number (percent) of predicted CL’_H_ that fell within 2-fold of the observed CL_H_ were 101 (96.2%), 79 (75.2%), 90 (85.7%), 85 (81.0%), and 103 (98.1%) for coumarin, paclitaxel, dextromethorphan, chlorzoxazone, and midazolam respectively.

Published correlations between different CYP gene and protein expression values could support the models for c_max_ in the present study [17], because the turnover rate is affected by the number of enzyme binding sites, which is determined by protein expression levels. K_m_, on the other hand, is about the affinity between substrates and binding sites, which is determined by enzyme and substrate structures. There is no published correlation between structures of CYPs from different chromosomes, although haplotypes exist for some CYPs on the same chromosome. As a consequence of this, predictive models for K_m_ of some CYPs could not be developed.

Although most previous studies have focused on *in vitro*–*in vivo* extrapolation of metabolic clearance in humans from hepatocyte or HLMs data [19,23,52,53], to our best knowledge, the present study is the first to try to establish descriptive models using a multiple linear regression method and attempt to combine the descriptive models and the CBC-IVIVE to extrapolate drug clearance *in vivo*. Encouragingly, we found that the predicted CL’_H_ values matched most closely with their clearance *in vivo.* To further validate the method established in the present study and the predicted results, the values of predicted CL_H_ for all five drugs were extrapolated based on measured CL_int_, which was used for comparative purposes. The results show that the values of predicted CL’_H_ were closer to the observed ones than those of predicted CL_H_. In addition, the percentage of predicted CL’_H_ that fell within 2-fold of the observed CL_H_ for all five drugs were greater than that of the predicted CL_H_.

Although these descriptive models could use data on some CYPs to predict activities of others, there are limitations. For example, different probe substrates could result in different observations in CYP3A4 activities due to different substrate binding sites [54]. In the present work, a single substrate was used to reflect the activity of each CYP. Therefore, the models might be applied only to the prediction of activities of substrates which were similar to probe substrates used in the present study. Another limitation is the representativeness of the sample. Although the models were established using the data on ten CYPs from 105 normal liver tissue samples from a Chinese Han population, the models should be further improved and validated in larger samples and in different ethnic groups.

In summary, we developed descriptive models to predict the activities of six CYPs (CYP2A6, 2C8, 2D6, 2E1, and 3A4/5) in HLMs by actually measuring the activities of four CYPs (CYP1A2, 2B6, 2C9, and 2C19). To be helpful for drug development, we combined the descriptive models and the CBC-IVIVE further to extrapolate the CL_H_ of probe drugs for six CYPs. While this approach has some limitations, it does establish a feasible method that can then be evaluated by additional experimental approaches and in additional populations. These findings may be of benefit for the development of personalized medicine and should be of significant value for drug development.

## Supporting information

**Supplemental Table S1:** The analytical methods for the measurement of substrate metabolites for the 10 CYP activity assays

## Abbreviations

AFE: average fold-error
BW: body weight
CC: correction coefficient
CBC-IVIVE: conventional bias corrected *in vitro–in vivo* extrapolation
CYP: cytochrome P450 enzyme
CL_H_: clearance *in vivo*
CL_int_: intrinsic clearance
CV: coefficient of variation
f_u,p_: plasma unbound fraction
HLM: human liver microsome
IVIVE: *in vitro*–*in vivo* extrapolation
IFE: individual fold-error
K_m_: Michaelis–Menten constant
LW: liver weight
MPPGL: microsomal protein per gram of liver
R_B_: blood-to-plasma concentration ratio
*R*^2^: coefficient of determination
*R*^2^_ad_: adjusted coefficient of determination
Q_H_: cardiac output
V_max_: maximum reaction rate
