# Supplementary material for: Prediction of cytochrome P450-mediated drug clearance in humans based on the measured activities of selected CYPs
Source: Biosci Rep. 2017 Nov 21;37(6):BSR20171161. doi: 10.1042/BSR20171161 (PMC5696450; doi:10.1042/BSR20171161)
Supplement: Supplementary file 1 [file bsr20171161_Supp1.pdf]

Supplementary Material

Prediction of cytochrome P450-mediated drug clearance in humans based on the measured activities of selected CYPs

Jie Gao[1], Jie Wang[1], Na Gao[1], Xin Tian[1], Jun Zhou[1], Yan Fang[1], Hai-Feng Zhang[1], Qiang Wen[1], Lin-Jing Jia[1], Dan Zou[2,*], Hai-Ling Qiao[1,*]

[1]Institute of Clinical Pharmacology, Zhengzhou University, Zhengzhou, China
[2]Department of Histology and Embryology, Henan Medical College, Zhengzhou, China

*Corresponding author
Hai-Ling Qiao. Email: qiaohl@zzu.edu.cn
Dan Zou. Email: zd6986@sina.com

The supplementary data contains Supplementary Table S1.

Supplementary Table S1. The analytical methods for the measurement of substrate metabolites for the 10 CYP activity assays

CYPs	Substrate	Metabolite	Determine wavelength (nm)	Mobile phase (V/V)
CYP1A2	Phenacetin	Acetaminophen	257	methanol/water (55/45)
CYP2A6	Coumarin	7-OH-coumarin	Ex=338, Em=458	methanol/perchloric acid (55/45)
CYP2B6	Bupropion	4-OH-bupropion	214	acetonitrile/50mM monopotassium phosphate (20/80)
CYP2C8	Paclitaxel	6-OH-paclitaxel	229	methanol/ammonium acetate (27/73)
CYP2C9	Tolbutamide	4-OH-tolbutamide	230	acetonitrile/0.03% phosphoric acid (34/66)
CYP2C19	Omeprazole	4-OH-omeprazole	302	acetonitrile/0.01M phosphate buffer (25/75)
CYP2D6	Dextromethorphan	3-methoxymorphinan	Ex=280, Em=320	phosphoric acid/ acetonitrile (70/30)
CYP2E1	Chlorzoxazone	6-OH-chlorzoxazone	287	methanol/water (55/45)
CYP3A4/5	Midazolam	1-OH-midazolam	220	methanol/acetic acid (66/34)

Diamonsil C_{18} column (200 mm×4.6 mm, 5 μm) for analyzing acetaminophen, 7-OH-coumarin, 4-OH-bupropion, 6-OH-paclitaxel, 4-OH-tolbutamide, and 6-OH-chlorzoxazone. Diamonsil C_{18} column (250 mm×4.6 mm, 5 μm) for 4-OH-omeprazole, 3-methoxymorphinan, and 1-OH-midazolam. V: volume; Ex: excitation wavelength; Em: emission wavelength.
